# The Anti-Inflammatory Effect of Multi-Wavelength Light-Emitting Diode Irradiation Attenuates Dry Eye Symptoms in a Scopolamine-Induced Mouse Model of Dry Eye

**DOI:** 10.3390/ijms242417493

**Published:** 2023-12-14

**Authors:** Hyeyoon Goo, Yea-Jin Lee, Sangkeun Lee, Namgue Hong

**Affiliations:** 1 Department of Medical Laser, Graduate School of Medicine, Dankook University, Cheonan 31116, Republic of Korea; ghy1204@hanmail.net; 2 Beckman Laser Institute Korea, Dankook University, Cheonan 31116, Republic of Korea; yjlee0109@dankook.ac.kr; 3 MEDI-IOT Co., Ltd., Seoul 02708, Republic of Korea; sklee.mediiot@gmail.com

**Keywords:** dry eye, multi-wavelength LED, photobiomodulation, cornea, pro-inflammatory cytokine, scopolamine

## Abstract

Dry eye disease is a common condition in patients of all ages, causing discomfort and potential visual problems. Current treatments, including artificial tears and anti-inflammatory drugs, have certain limitations, encouraging research into alternative therapies. We investigated the therapeutic potential of multi-wavelength light-emitting diode (LED) irradiation of mice with dry eye. First, we showed that multi-wavelength LED irradiation was non-toxic to human corneal epithelial cells and improved cell viability. We then used a scopolamine-induced mouse model of dry eye to assess the effects of multi-wavelength LED irradiation on various clinical parameters. This treatment increased the tear volume and reduced corneal irregularity, thus improving dry eye. Histological analysis revealed that multi-wavelength LED irradiation protected against corneal epithelial damage and the associated reduction in epithelial thickness and would thus improve the corneal health of dry eye patients. Multi-wavelength LED irradiation significantly reduced the corneal levels of pro-inflammatory cytokines IL-6, IL-1β, and TNF-α; the treatment was thus anti-inflammatory. Our results suggest that multi-wavelength LED irradiation may serve as a safe and effective treatment for dry eye, alleviating symptoms, reducing inflammation, and promoting corneal health.

## 1. Introduction

Dry eye is a chronic multifactorial disease of the ocular surface common in patients of all ages worldwide [1,2]. The condition is characterized by irritated, gritty, scratchy, or burning eyes that can later exhibit corneal surface irregularities that decrease vision and increase the risk of corneal ulcers [3,4,5,6]. In dry eye, the osmotic pressure of the tear film increases, triggering irritation, inflammation, and secretion of inflammatory mediators [7]. As the corneal surface becomes damaged, additional inflammatory cytokines are produced by activated cells, triggering a chain reaction [8,9]. Treatment depends on the cause and symptoms; topical medications are currently the most effective therapeutics, but long-term use is associated with toxicity, increased intraocular pressure, and cataracts [10]. The main goal is to control inflammation.

Recently, light-emitting diode (LED)-based photobiomodulation (PBM) has been used to treat many inflammatory diseases, including neurological and dermatological conditions; several researchers are exploring PBM in an ophthalmological context [11,12,13,14,15]. PBM phototherapy uses visible and near-infrared (NIR) light to control pain and inflammation without any complications, aiding immune system regulation, tissue production, wound healing, and neurogenesis [16,17,18]. In particular, red LED light with a wavelength of 600–700 nm enhances the immune response and cell proliferation [19,20], and NIR LED light with a wavelength 800 nm exerts anti-inflammatory effects [21,22]. Several studies reported that PBM effectively improved the tear breakup time and tear volume and reduced the inflammatory response [23,24,25,26]. Several clinical studies have explored the effects of LED light on the ocular surfaces of dry eye patients; the treatment was useful, but the parameters studied varied [24,27,28]. Although single-wavelength LEDs have been extensively studied, multi-wavelength LEDs have not. Therefore, we evaluated the in vivo effects of simultaneous 680, 780, and 830 nm LED light treatments of mice with dry eye.

## 2. Results

### 2.1. Multi-Wavelength LED Irradiation Has No Toxicity on Human Corneal Epithelial Cells

**Figure 1:**
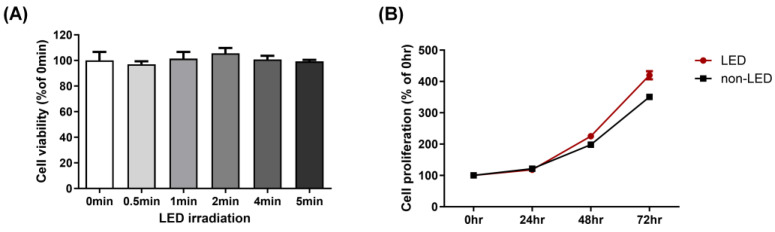
Effects of multi-wavelength LED on cell viability and cell proliferation of human corneal epithelial (HCE-T) cells. (**A**) Cell viability was measured 24 h after exposing the cells to LED irradiation at an intensity of 20 mW/cm^2^ for varying durations (0, 0.5, 1, 2, 4, and 5 min). (**B**) After LED irradiation at 20 mW/cm^2^ for 2 min; cell proliferation of the LED and non-LED samples for 24, 48 and 72 h. Data are the means ± SEM (*n* = 3).

While LED light itself is not inherently harmful to the eyes, prolonged exposure to the blue light emitted by LED lighting can lead to eye fatigue, headaches, and other adverse effects on vision. Therefore, it is necessary the assess the safety of the multi-wavelength LED at the cellular level. First, we used the MTT assay to explore whether multi-wavelength LED irradiation is toxic to human corneal HCE-T cells. No toxicity was observed after irradiation at 20 mW/cm^2^ for 0, 0.5, 1, 2, 4, and 5 min. Indeed, after multi-wavelength LED irradiation for 2 min, cell viability increased, albeit not significantly, compared to the no-irradiation sample (0 min) (Figure 1A). We evaluated cell viability from 0 to 72 h after 2 min of multi-wavelength irradiation. Figure 1B shows the viability of HCE-T cells after irradiation with multi-wavelength LED light of 20 mW/cm^2^ for 0, 24, 48, and 72 h; no cytotoxicity was apparent. Indeed, at 72 h, cell viability was greater than that of non-irradiated (non-LED) cells. Multi-wavelength LED irradiation for 2 min was thus employed in the in vivo experiments described below.

### 2.2. Effect of Multi-Wavelength LED Irradiation on the Clinical Features of Scopolamine-Induced Dry Eye

**Figure 2:**
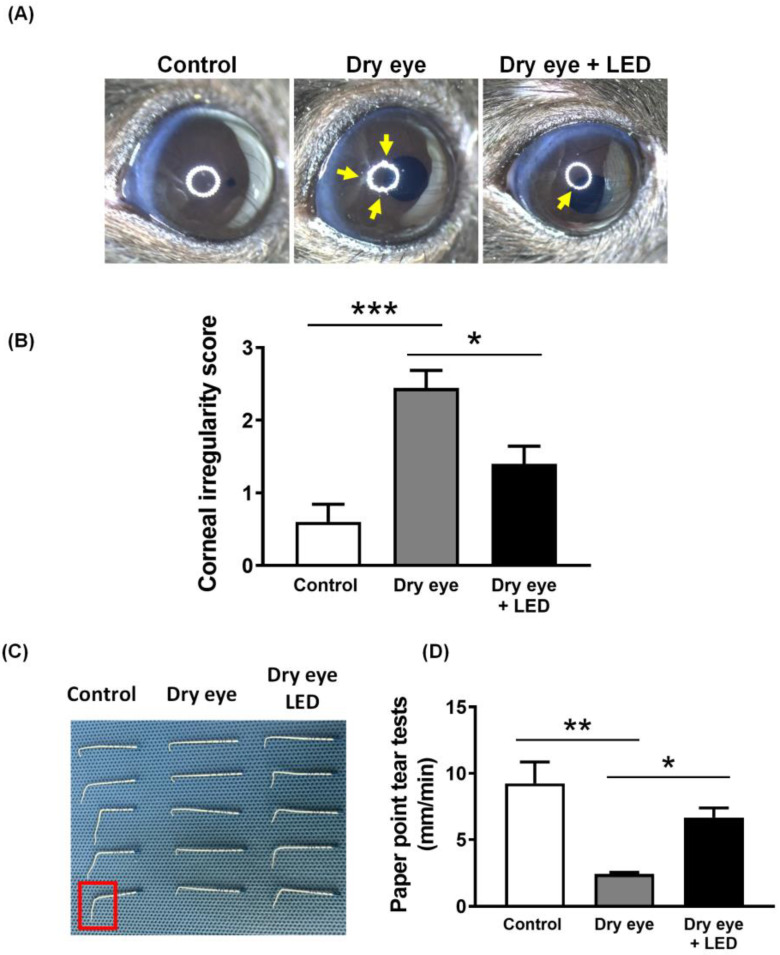
Effects of multi-wavelength LED on tear production and corneal surface irregularities in the dry eye mice. (**A**) Representative image of the corneal surface taken with a stereoscopic zoom microscope (Yellow arrow; distorted white rings). (**B**) Corneal irregularities were scored on day 17 of dry eye induction (score: 0 = no damage, 1 = slightly irregular, 2 = irregular less than 50% circle, 3 = irregular more than 50% circle). (**C**) Representative photograph by group demonstrating how the absorption of the aqueous fraction of the tear was measured in the standardized endodontic absorbent paper points. (**D**) Quantitative graph measuring the end portion of the paper point moistened with tears (Red square, paper point moistened with tears). Data are the means ± SEM (Control: *n* = 5, Dry eye: *n* = 5, Dry eye + LED: *n* = 5). * *p* < 0.05, ** *p* < 0.01, *** *p* < 0.001 compared to the group induced to dry eye (one-way ANOVA with the Bonferroni post hoc test).

PBM is already gaining attention as a potential therapeutic approach for various ocular conditions (11, 15, 25). However, the evidence for improvement in dry eye symptoms through PBM in an animal model is still quite limited, and much research is needed to substantiate these potentials. First, to investigate whether multi-wavelength LED irradiation ameliorated dry eye, we treated all eyes for 2 min per day on five successive days. In Figure 2, scopolamine-induced the clinical features of dry eye, i.e., greater corneal irregularity than in the control group (score = 2.38 ± 0.25 (*n* = 8) and 0.60 ± 0.22 (*n* = 8), respectively) and decreased tear volume (2.42 ± 0.11 and 9.25 ± 1.44 mm, respectively). Multi-wavelength LED irradiation significantly reduced corneal irregularity (score 1.40 ± 0.22, *n* = 8) and increased the tear volume (6.67 ± 0.65 mm, *n* = 8).

### 2.3. Effect of Multi-Wavelength LED Irradiation on Corneal Epithelia

**Figure 3:**
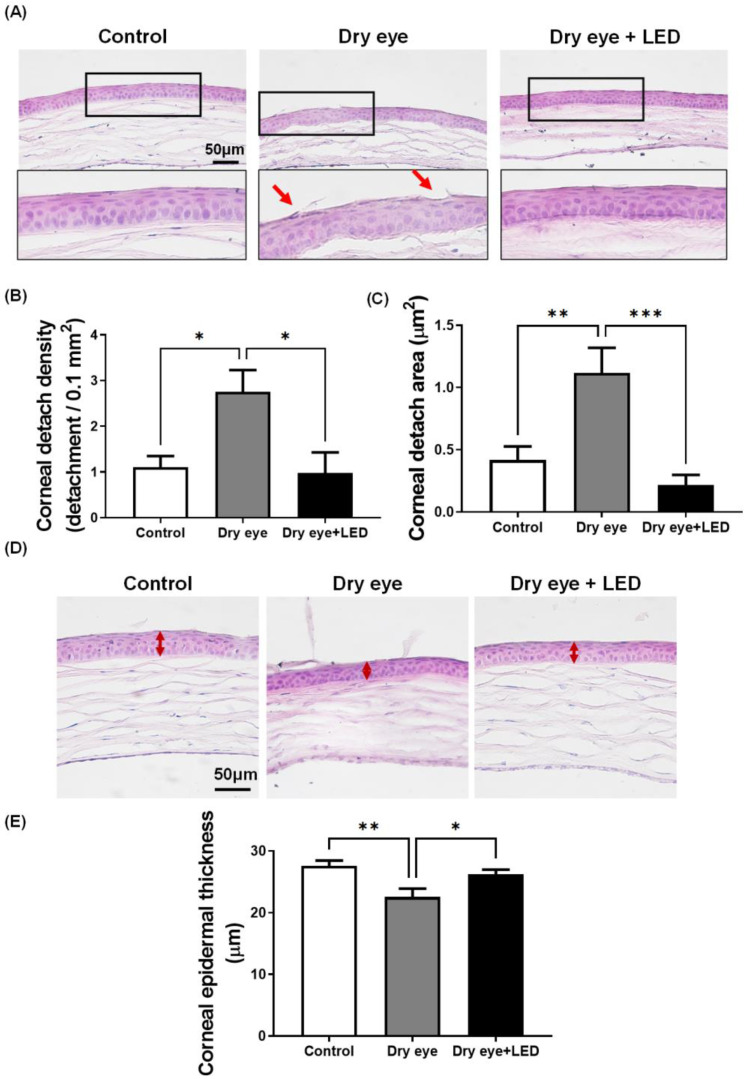
Effects of multi-wavelength LED on damaged corneal epithelium of dry eye mice. (**A**) The corneas of mice treated with LED for 5 days to induce dry eye for 17 days were stained with H&E. Red arrows, detached corneal epithelial cells; Black square, location of the magnified figures below. (**B**) Quantitative graph measuring the corneal detachment epithelial cell and (**C**) corneal detachment area. Representative photograph (**D**) and quantitative graph (**E**) by group demonstrating the effect of LED treatment on epidermal thickness. Bidirectional arrow, epidermal thickness. Scale bars are indicated in each image. Data are the means ± SEM (Control: *n* = 5, Dry eye: *n* = 5, Dry eye + LED: *n* = 5). * *p* < 0.05, ** *p* < 0.01, *** *p* < 0.001 compared to the group induced to dry eye (one-way ANOVA with the Bonferroni post hoc test).

To investigate whether scopolamine-induced dry eye affected the corneal epithelium and whether multi-wavelength LED irradiation modulated such an effect, we stained corneas with hematoxylin and eosin. Figure 3 shows that cell detachment and reduced corneal epithelial area were more common in the dry eye group than in the controls, but LED irradiation protected against such changes. Detached cell numbers/0.1 mm^2^ are provided. Dry eye increased corneal epithelial cell detachment (3.09 ± 0.41 cells) and the affected area (1.26 ± 0.22 µm^2^). However, in the dry eye + LED group, the number of detached cells (0.95 ± 0.33) and the affected area (0.20 ± 0.07 µm^2^) were significantly decreased. The corneal epithelium of the control group included five to six layers of cells with a total thickness of 27.87 ± 0.38 µm. The dry eye group had fewer layers and the thickness was significantly lower, at 21.54 ± 0.49 µm. The thickness for the dry eye + LED group was 26.34 ± 0.39 µm, which was significantly greater than that of the dry eye group. Thus, multi-wavelength LED irradiation protected the corneal epithelium.

### 2.4. Effect of Multi-Wavelength LED Irradiation on Pro-Inflammatory Cytokine Levels

**Figure 4:**
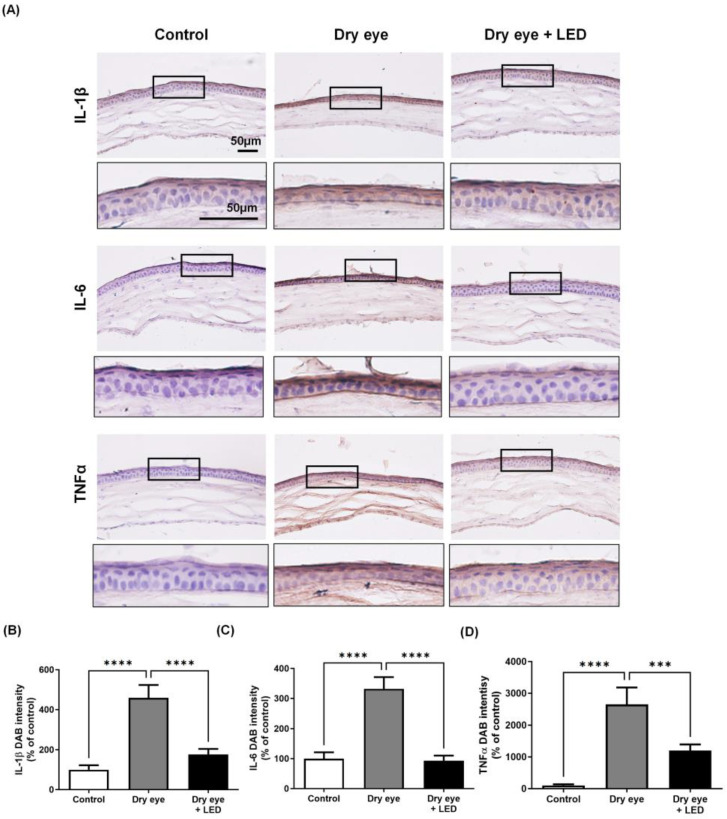
Effects of multi-wavelength LED on pro-inflammatory markers in the corneal of dry eye mice. (**A**) The corneas of mice treated with LED for 5 days to induce dry eye for 17 days were immunostained with specific antibodies for IL-1β, IL-6, and TNF-α. Immunostaining was performed using DAB and is expressed in brown. Black square, location of the magnified figures below. (**B**–**D**) The stained area of the photograph was analyzed using ImageJ and the intensity of the expressed area was calculated as intensity. Scale bar= 50μm. Data are the means ± SEM (Control: *n* = 5, Dry eye: *n* = 5, Dry eye + LED: *n* = 5). *** *p* < 0.001, **** *p* < 0.0001 compared to the group induced to dry eye (one-way ANOVA with the Bonferroni post hoc test).

To investigate whether the protective effects of multi-wavelength LED on corneal epithelial damage are associated with the inflammatory response, a key factor in dry eye syndrome, we examined several inflammatory cytokine markers. The pro-inflammatory markers TNFα, IL-6, and IL-1β were immunohistochemically stained brown with DAB and the extent of staining was quantitated using ImageJ software (1.52n ver.; NIH, Bethesda, MD, USA). Figure 4 shows the immunostained corneal sections. The IL-1β, IL-6, and TNF-α levels of the dry eye group (459.2 ± 63.3, 332.01 ± 38.36, and 2646.96 ± 518.68%, respectively) were significantly higher than those of the control group (100 ± 1.0, 100 ± 20.80, and 100 ± 0.21%, respectively), but these levels were significantly decreased in the dry eye + LED group (176.74 ± 31.86, 93.01 ± 16.97, and 1202.30 ± 190.41%, respectively) compared to the dry eye group. To validate the DAB image results, we observed the expression of pro-inflammatory cytokines using fluorescent secondary antibodies (Figure S1). The expression of pro-inflammatory cytokines was evaluated by analyzing the expressed fluorescence intensity with ImageJ software (1.52n ver.; NIH, Bethesda, MD, USA). Immunofluorescent staining results also indicated an increase in inflammatory cytokines such as IL-1β, IL-6, and TNFα in the dry eye group, while the dry eye + LED group exhibited an overall significant decrease. Thus, multi-wavelength LED irradiation triggered histological changes.

## 3. Discussion

Dry eye is a multifactorial ocular surface condition characterized by loss of tear film homeostasis and other ocular symptoms. Tear film instability and hyperosmolarity, ocular surface inflammation and damage, and neurosensory abnormalities play causal roles [29]. Artificial tears, anti-inflammatory drugs, and autologous serum are used to treat dry eye. However, artificial tears only temporarily relieve symptoms; anti-inflammatory drugs can trigger serious long-term side effects, and autologous serum can cause infections [30]. PBM is an acronym for phototherapy employing red and near-infrared LEDs, used by dermatologists and other medical professionals, which has no side effects. PBM promotes tissue and nerve regeneration, reduces pain and inflammation, and enhances tissue repair [31,32]. Few studies have used red and near-infrared LEDs to treat ocular tissue. Goo et al. reported that low-level light (740 nm) increased tear volume in an animal model of dry eye; Park et al. reported that 590 and 830 nm LED treatments were effective in dry eye patients [33,34]. Here, we explored the in vivo effects of LED irradiation at 680, 780, and 830 nm on mouse dry eye.

After dry eye was induced by scopolamine, the tear volume and corneal surface irregularity were evaluated; these parameters are commonly used to both diagnose dry eye and monitor disease progression [35]. Clinically, the multi-wavelength LED irradiation group improved compared to the dry eye group. The tear volume significantly increased and corneal surface irregularity significantly decreased. LED NIR light increases the migration capacity of corneal epithelial cells [30], and 600 nm red light enhances wound healing of the corneal epithelium [36]. We found that corneal epithelial cell detachment increased in the dry eye group but was reduced after multi-wavelength LED irradiation.

The levels of some pro-inflammatory cytokines were measured as markers of the extent of inflammation. IL-6, IL-1β, and TNF-α play important roles in ocular inflammation; dry eye increases the levels thereof [30,37]. Remarkably, we found that the levels of IL-6, IL-1β, and TNF-α increased in the corneas of dry eyes but significantly decreased after multi-wavelength LED treatment; these cytokines thus contribute to dry eye inflammation [38,39]. The effects of intra-ocular LED treatment on pro-inflammatory cytokine levels remain unknown, although some studies on other tissues reported that LED light reduced and controlled inflammation [40,41].

Multi-wavelength LED irradiation not only reduced the levels of inflammatory cytokines in dry eye corneal tissue but also afforded clinical improvement. However, this study had several limitations. First, only corneal tissue was collected; the conjunctiva and lacrimal glands were not studied. Dry eye commonly affects the cornea [42], but the lacrimal gland and conjunctiva protect against infection and inflammation and maintain corneal transparency [43,44]; the effects of LED light on these tissues require evaluation. Additionally, the levels of some pro-inflammatory cytokines were not assayed. Inflammation involves various chemokines and the actions of macrophages, neutrophils, and T cells [45]. It remains unknown whether multi-wavelength light reduces these reactions; this requires further study. Finally, although many researchers have studied the effects of LED light, the parameters that they evaluated differed. Multi-wavelength LED light effectively treats dry eye; the effects of the individual wavelengths should be explored. The optimal LED wavelength and power remain unknown and should be determined to ensure effective treatment.

Various clinical studies are currently underway to investigate the use of PBM for the treatment of dry eye. A clinical trial involving 40 patients revealed the safety and significant improvement of PBM [34]. Despite these favorable outcomes, there is inadequate support from asymptomatic animal studies. In their clinical analysis, the authors assert that PBM-mediated inflammation control targeting ocular surface inflammation in dry eye patients provides evidence for improving dry eye as a contributing factor to visual deterioration [46]. Most PBM studies have predominantly employed single-wavelength LED for efficiency [34,47]. Therefore, our present study is essential as a foundation for future clinical trials investigating multi-wavelength LED applications. The study has some limitations, including the absence of a clear in vitro demonstration of PBM’s pathogenic mechanism. It would have been preferable to include intricate cellular experiments to elucidate detailed mechanisms in corneal cells. Another limitation is the existing disparity between animal models and clinical environments. Clinically, dry eye is often attributed to prolonged exposure to environmental factors in human life, hence exposing animal models to conditions inducing dry eye over a period of 3–6 months would better align with the experimental context. As mentioned earlier, this represents the sole animal study employing multi-wavelength LED to demonstrate beneficial effects for dry eye. The study provides evidence supporting the efficacy of multi-wavelength LED in treating dry eye and, notably, furnishes histological results not feasible in a clinical trial setting.

In conclusion, this is the first study to explore whether multi-wavelength LED irradiation improved dry eye in an animal model. The irradiation increased the tear volume, reduced corneal irregularities, enhanced epithelial integrity, and reduced the inflammatory response. Multi-wavelength LED irradiation may be useful to treat human dry eye syndrome.

## 4. Materials and Methods

### 4.1. LED Treatment

**Figure 5:**
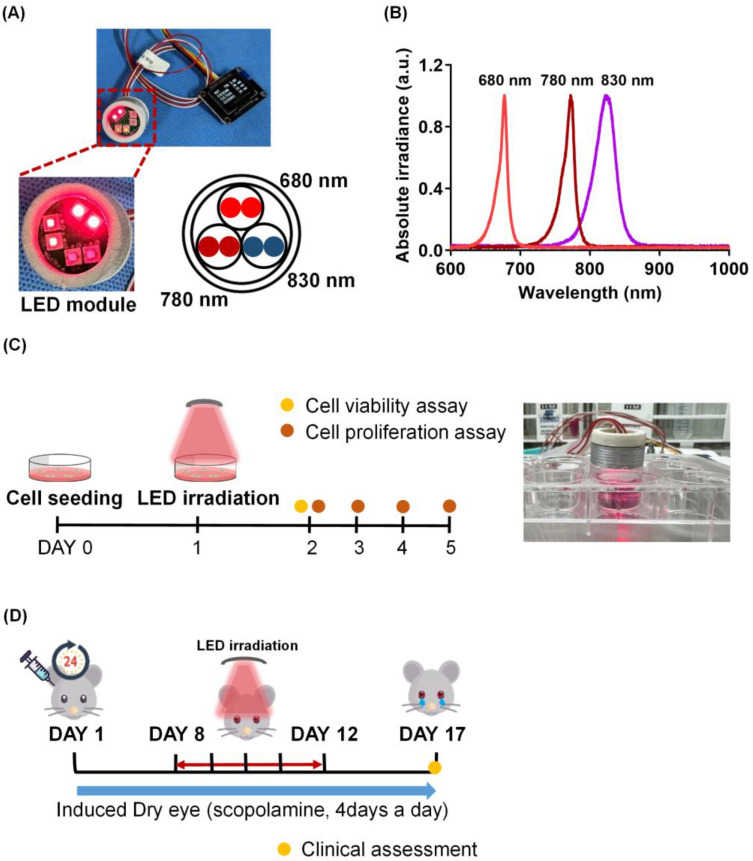
LED irradiance and experimental schedule for the in vivo and in vitro studies. (**A**) Experimental LED irradiation is illustrated including images of multi-wavelength LED panels. The detailed parameters of LED irradiation are shown in Table 1. (**B**) Multi-wavelength LED irradiation was measured using a spectrometer. (**C**) For in vitro studies, human corneal epithelial cells (HCE-T) were seeded and irradiated with LED according to conditions 24 h later (DAY 1). After the irradiation, cell viability (DAY 2) and cell proliferation (DAY 2, 3, 4, 5) were analyzed. (**D**) C57/BL6 7-week-old mice were used for the in vivo study. To induce dry eye, scopolamine was injected subcutaneously four times daily for 17 days, and the eyes were irradiated with LED daily for 5 days starting from the 8th day after the experiment. The tear volume and corneal irregularity score of each mouse were measured on the 17th day of the experiment.

The light source was a multi-wavelength LED; the wavelengths were 680 (EDC680D-1100), 780 (EDC780D-1100), and 830 nm (EDC830N-1100) (Figure 5A). LED light was measured using a spectrometer (USB4000; Ocean Optics, Orlando, FL, USA; Figure 5B). The LED chip dimensions were 1000 × 1000 µm, and the LEDs were mounted in a 3.5 × 3.5 mm ceramic package. LED power was measured using a laser fiber spectroradiometer combined with a diffuser. Detailed laser data, including the full width at half-maximum and LED electrical power values are shown in Table 1.

### 4.2. Cell Viability and Proliferation Assays

The human corneal epithelial cell line HCE-T was purchased from ATCC (Manassas, VA, USA) and grown in Dulbecco’s modified Eagle’s medium (DMEM) supplemented with 1% (*w*/*v*) penicillin and streptomycin (Gibco, Grand Island, NY, USA) and 5% (*v*/*v*) heat-inactivated fetal bovine serum (FBS; Equitech-Bio Inc, Kerrville, TX, USA). Cells were maintained at 37 °C under 5% (*v*/*v*) CO_2_ in a humidified chamber. Cells were exposed to LED light at 0.6, 1.2, 2.4, 4.8, and 6 J/cm^2^ followed by incubation and evaluation of cell viability. Cell proliferation was examined after LED treatment at 2.4 J/cm^2^ followed by incubation for 0, 24, 48 and 72 h. After incubation, 150 μL of 2,5-diphenyl-2H-tetrazolium bromide (MTT) solution (5 mg/mL) was added to each well, and 4 h later, the medium was removed and 100 μL dimethyl sulfoxide (DMSO) was added to each well to dissolve the violet blue crystals. Cell growth was determined by measuring absorbance at 570 nm using an ELISA reader (Synergy HTX; BioTek, Santa Clara, CA, USA).

### 4.3. Mouse Model of Dry Eye and the Experimental Procedure

The research protocol was approved by the Dankook University Medical School Research Institutional Animal Care and Use Committee (approval no. DKU-23-019). All animals were treated in accordance with the ARVO Statement for the Use of Animals in Ophthalmic and Vision Research. We used 8-week-old female C57BL/6 mice. Dry eye was induced via four subcutaneous injections of 2.5 mg/mL scopolamine hydrobromide (Sigma-Aldrich, St. Louis, MO, USA) at 09.00, 12.00, 15.00, and 18.00. Fifteen mice were randomly assigned to three groups: untreated (control), dry eye induced by scopolamine (Dry eye), and mice with dry eyes irradiated with the LED (Dry eye + LED). After 7 days, LED radiation was delivered at the time of dry eye induction and repeated over the next 4 days. Two clinical assessments were conducted; we measured tear volumes and corneal irregularities. At the end of the experiment, eyeballs were removed and stained.

### 4.4. Tear Volume Test

Standard absorbent paper (Roeko Color 30; COLTENE DE, Langenau, Germany) was used to assess tear volume. Each mouse was held in one hand and the tip of the paper was inserted into the lateral canthus with the other hand; the paper remained in position for 1 min. When moistened with tears, the paper bends when laid on a hard surface; the distance between the end of the paper and the bending point was measured in mm [48].

### 4.5. Evaluation of Corneal Damage

To assess corneal surface irregularities, images of white rings on corneal surfaces were acquired using a stereoscopic zoom microscope (Nikon, Tokyo, Japan) immediately after three induced blinks. Corneal surface irregularities (corresponding to poor corneal integrity) caused by UV radiation presented as distortions in the reflected rings that were classified as nil (0), few irregularities (1), or irregularities over < or ≥ 50% of the ring (2 and 3, respectively).

### 4.6. Histology

Eyeballs were removed from mice, fixed in 4% (*v*/*v*) formaldehyde (Daejung, Siheung, Republic of Korea), frozen, and sectioned using a cryostat. To preserve tissue morphology, eyeball tissues were fixed in 4% (*v*/*v*) formaldehyde overnight at 4 °C, transferred to 15% (*w*/*v*) sucrose (Sigma-Aldrich) for 4 h at 4 °C, and transferred to 30% (*w*/*v*) sucrose overnight at 4 °C. OCT embedding compound (Sakura, Tokyo, Japan) was then added and tissues were frozen at −20 to −80 °C. Tissue sections (10 µm) were prepared using a cryostat, thaw-mounted onto gelatin-coated slides, and stained with Harris hematoxylin solution for 5 min; 1% (*v*/*v*) alcohol was added after deparaffinization. The sections were rinsed with tap water, counterstained in eosin Y solution for 2 min, and observed under a microscope (BX53; Olympus, Tokyo, Japan). Corneal detachment and epidermal thickness were quantified using ImageJ software (1.52n ver.; NIH, Bethesda, MD, USA).

### 4.7. Immunohistochemistry

Immunohistochemical analysis (IHC) was performed as described previously. Slides were dried on a warming block at 37 °C. All sections were washed twice with 1× PBS for 5 min each time, blocked with 5% (*w*/*v*) BSA in 1× PBS for 1 h, incubated with primary antibodies against IL-6, IL-1β and TNF-α overnight at 4 °C, washed twice for 5 min each time in 1× PBS, and incubated with goat-anti rabbit biotinylated secondary antibody (Vector Laboratories Inc., Burlingame, CA, USA) (1:500 in blocking buffer) for 1 h at room temperature. All sections were then treated with ABC solution (Vectastain ABS Elite kit; Vector Laboratories Inc.) for 1 h, washed with 1× PBS, and incubated with 3,3′-diaminobenzidine (DAB) solution from a peroxidase substrate kit (Vector Laboratories Inc.) for 20 s. Counterstaining was performed using Meyer’s hematoxylin (Dako, Carpinteria, CA, USA). Stained sections were viewed under a microscope (Nikon, Tokyo, Japan) and intensity was quantified using ImageJ software (1.52n ver.; NIH, Bethesda, MD, USA).

### 4.8. Statistics

All data are expressed as the mean  ±  standard deviation of the mean (SEM), and data were analyzed using GraphPad Prism Software (ver. 10.0.2; La Jolla, CA, USA). The Shapiro–Wilk test was used to determine whether data were normally distributed. One-way analysis of variance (ANOVA) with the Dunn multiple comparisons test as well as two-way ANOVA with Bonferroni post hoc analysis were used to reject the null hypothesis when comparing more than two groups. *p*  <  0.05 was considered statistically significant. * *p* < 0.05, ** *p* < 0.01, *** *p* < 0.001, and **** *p* < 0.0001.

## Figures and Tables

**Table 1:** Specifications for the multi-wavelength LED parameters.

Treatment Wavelength (nm)	680 nm	780 nm	830 nm
Light type	Light-emitting diode		
Chip dimension	1000 μm × 1000 μm		
Mode	Continuous wave (CW)		
FWHM	22 nm	25 nm	35 nm

## Data Availability

The data that support the findings of this study are available from the corresponding author upon reasonable request.

## Supplementary Materials

The following supporting information can be downloaded at: https://www.mdpi.com/article/10.3390/ijms242417493/s1.
