# Supplementary material for: The Anti-Inflammatory Effect of Multi-Wavelength Light-Emitting Diode Irradiation Attenuates Dry Eye Symptoms in a Scopolamine-Induced Mouse Model of Dry Eye
Source: Int J Mol Sci. 2023 Dec 14;24(24):17493. doi: 10.3390/ijms242417493 (PMC10744116; doi:10.3390/ijms242417493)
Supplement: Supplementary file 1 [file ijms-24-17493-s001.zip › ijms-2682064-supplementary.pdf]

Supplementary file

Supplementary file 1: Figure of fluorescent intensity analysis of pro-inflammatory cytokine markers

Supplementary figures

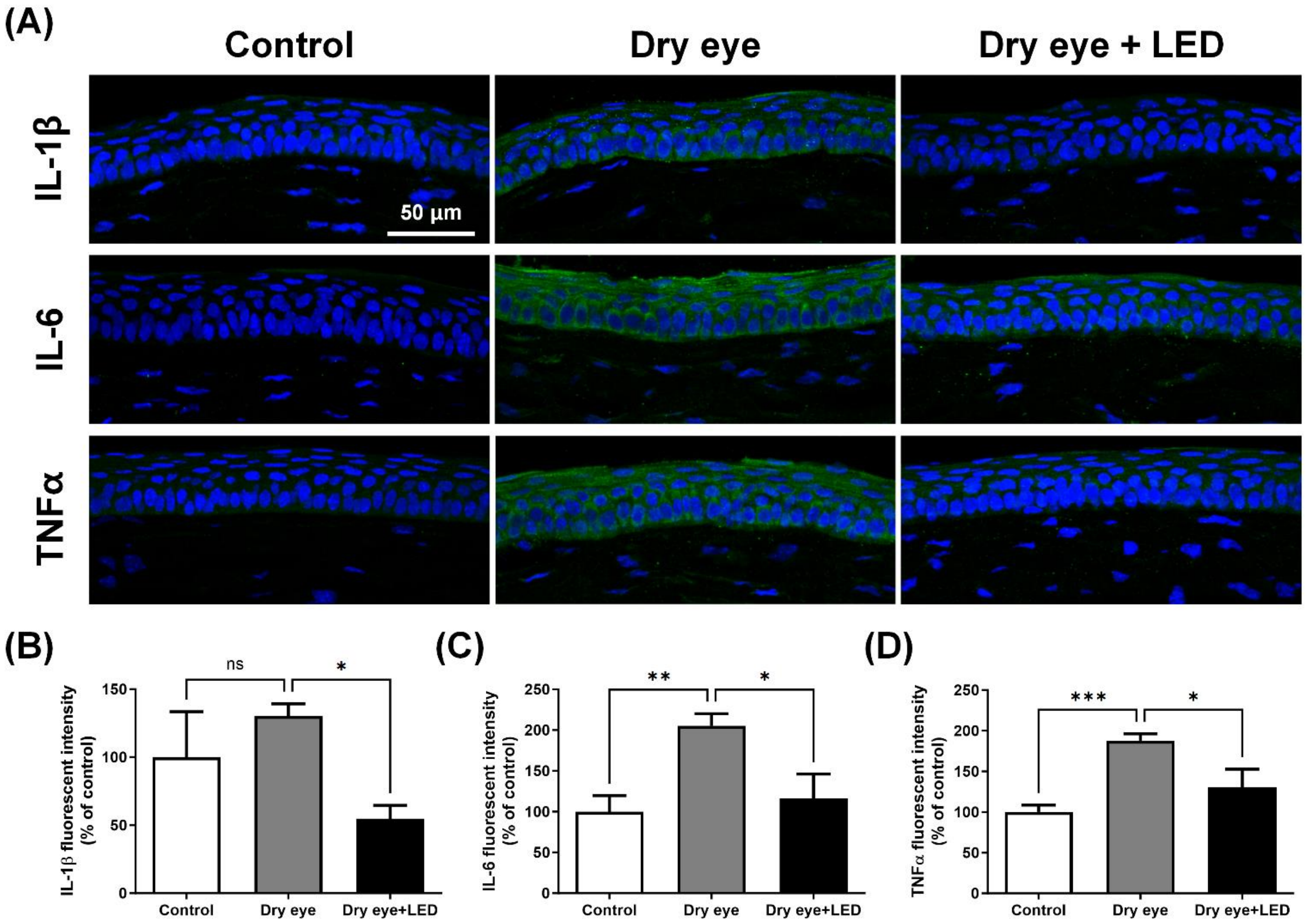

Figure S1. Multi-wavelength LED attenuated the expression of pro-inflammatory markers in the corneal epithelium of dry eye mice. (A) Representative images of immunostained IL-1β, IL-6 and TNF-α (green) showed the expression pattern of pro-inflammatory cytokines in the corneal epithelium. The scale bar indicates 50 µm. (B-D) The fluorescent intensity of pro-inflammatory cytokines was quantified (IL-1β, IL-6 and TNF-α, respectively). This quantification revealed that the multi-wavelength LED significantly decreased the expression of pro-inflammatory markers in the corneal epithelium of dry eye. Data are expressed as the mean ± SEM; $^*p < 0.05$, $^{**}p < 0.01$, $^{***}p < 0.001$ relative to Dry eye group (one-way ANOVA with the Bonferroni post-hoc test).
